# Piezo1-mediated mechanohydraulic control of cell volume drives cardiac morphogenesis

**DOI:** 10.1126/sciadv.aea7025

**Published:** 2026-04-22

**Authors:** Christina Vagena-Pantoula, Konstantinos Kalyviotis, Shuyi Feng, Antoine Sanchez, Igor Kondrychyn, Moe Fukumoto, Xiangbin Pan, Thomas Juan, Didier Y. R. Stainier, Periklis Pantazis, Li-Kun Phng, Julien Vermot

**Affiliations:** ^1^ Department of Bioengineering, Imperial College London, London, UK.; ^2^ The Francis Crick Institute, London, UK.; ^3^ Randall Centre for Cell and Molecular Biophysics, School of Basic and Medical Sciences, Faculty of Life Sciences and Medicine, King’s College London, London, UK.; ^4^ Department of Structural Heart Disease, National Center for Cardiovascular Disease, China and Fuwai Hospital, Chinese Academy of Medical Sciences and Peking Union Medical College, Beijing, China.; ^5^ Laboratory for Vascular Morphogenesis, RIKEN Center for Biosystems Dynamics Research, Kobe, Japan.; ^6^ Department of Cell Biology, National Cerebral and Cardiovascular Center Research Institute, Suita, Osaka, Japan.; ^7^ Department of Immunology, Genetics and Pathology, Uppsala University, Uppsala, Sweden.; ^8^ Department of Developmental Genetics, Max Planck Institute for Heart and Lung Research, Bad Nauheim, Germany.; ^9^ German Centre for Cardiovascular Research (DZHK), Partner Site Rhine-Main, Bad Nauheim, Germany.; ^10^ Cardio-Pulmonary Institute (CPI), Bad Nauheim, Germany.

## Abstract

Organ morphogenesis is driven by physical forces, yet how mechanical stimuli pattern tissue shape and guide developmental programs remains poorly understood. In zebrafish, endocardial cells (EdCs) within the heart valve–forming region undergo marked volume reduction during early morphogenesis. Here, we uncover a hydraulics-based mechanism by which mechanical forces control EdC volume to direct cardiac development. We show that the mechanosensitive ion channel Piezo1 acts with the calcium-binding protein calmodulin (CaM) and the aquaporin Aqp8a.1 water channel to orchestrate EdC shrinkage. We find that Aqp8a.1 mediates cell volume loss by incorporating into the plasma membrane in response to mechanical stimulation, promoting heart looping and valve formation. Mechanistically, Piezo1 governs Aqp8a.1 through a dual mechanism. First, Piezo1 and CaM drive Aqp8a.1 plasma membrane incorporation, enabling rapid cell volume adjustments. Second, Piezo1 suppresses *aqp8a.1* transcription via Notch1b signaling to prevent excessive shrinkage. Together, these findings reveal that mechanotransduction can dictate organ formation through dynamic cell volume regulation, uncovering a fundamental principle of morphogenesis.

## Teaser

Mechanical forces shape the developing heart by controlling cell volume through a Piezo1-driven hydraulic mechanism.

## INTRODUCTION

Mechanical forces shape embryonic development by driving cell morphology, positioning, proliferation, and gene expression (*1*–*3*). These dynamic cues are integral to organogenesis, guiding cell fate decisions, orchestrating tissue patterning, and maintaining homeostasis (*1*–*3*). Among the cellular processes involved, cell hydraulics, the pressure-regulated transport of water across membranes, plays a central role (*4*–*9*). It enables precise volume regulation (*10*, *11*), allowing dynamic cell shape changes that ultimately define the architecture of emerging tissues (*4*–*9*). In the developing zebrafish heart, endocardial cells (EdCs) located in the valve-forming region are subjected to substantial mechanical forces due to blood flow and osmotic pressure arising from the extracellular matrix (*12*–*27*) and exhibit a significant decrease in volume during early stages (*14*). While increasing evidence suggests that mechanosensation and fluid transport are tightly coupled during morphogenesis, how mechanical stimuli regulate water flux and cell volume in vivo remains poorly understood (*4*, *14*, *28*, *29*).

Here, we identify a hydraulics-driven, force-responsive pathway that governs EdC volume regulation and drives cardiac morphogenesis. We show that the mechanically activated ion channel Piezo1 acts together with the calcium-binding protein calmodulin (CaM) and the aquaporin water channel Aqp8a.1 to mediate EdC shrinkage. Aqp8a.1 responds to mechanical cues by incorporating into the plasma membrane, facilitating water efflux and volume reduction. This response is coordinated with the volume-regulated anion channel (VRAC), which supports efficient water movement. Notably, Piezo1 regulates EdC volume through two distinct mechanisms: It promotes the rapid integration of Aqp8a.1 into the plasma membrane via CaM, and it represses *aqp8a.1* transcription through Notch1b signaling, thereby preventing excessive shrinkage. Collectively, these findings uncover a mechanosensitive signaling cascade that regulates water flux and cell volume to shape the developing heart.

## RESULTS

### Cell hydraulics controls EdC volume and cardiac morphogenesis

**Fig. 1.**
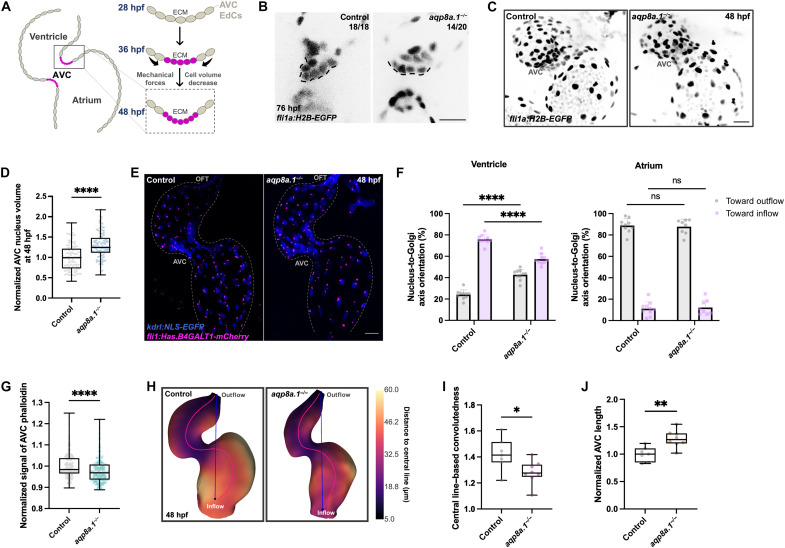
Aqp8a.1 is essential for AVC EdC volume reduction and heart development. (**A**) Schematic illustrating AVC EdC volume reduction. ECM, extracellular matrix. (**B**) Single *z*-plane confocal images of the superior AV valve leaflet in control (*n* = 18) and *aqp8a.1^−/−^* (*n* = 20) larvae in *Tg(fli1a:H2B-EGFP)* background at 76 hours postfertilization (two independent experiments). Dotted lines outline valve leaflet. Scale bar, 10 μm. (**C**) Confocal images of control and *aqp8a.1^−/−^* hearts in *Tg(fli1a:H2B-EGFP)* background at 48 hours postfertilization. Scale bar, 20 μm. (**D**) AVC nucleus volume in control (*n* = 91 nuclei/7 embryos) and *aqp8a.1^−/−^* (*n* = 105 nuclei/8 embryos) embryos in *Tg(fli1a:H2B-EGFP)* background at 48 hours postfertilization, normalized to control (Mann-Whitney test; *P* < 0.0001; two independent experiments). (**E**) Confocal images of control and *aqp8a.1^−/−^* hearts in *Tg(kdrl:NLS-EGFP);(fli1:Has.B4GALT1-mCherry)* background at 48 hours postfertilization. Dotted lines outline the endocardium. Scale bar, 20 μm. (**F**) Cell orientation percentages in control (*n* = 10) and *aqp8a.1^−/−^* (*n* = 10) embryos at 48 hours postfertilization [two-way analysis of variance (ANOVA); mean ± SD; both comparisons, *P* < 0.0001; two independent experiments]. (**G**) Phalloidin signal at the AVC in control [*n* = 245 regions of interest (ROIs)/7 embryos] and *aqp8a.1^−/−^* (*n* = 301 ROIs/8 embryos) embryos at 48 hours postfertilization, normalized to control (Mann-Whitney test; *P* < 0.0001; two independent experiments). (**H**) Three-dimensional (3D) visualizations of 48 hours postfertilization control and *aqp8a.1^−/−^* hearts. (**I**) Central line–based convolutedness in 48 hours postfertilization control (*n* = 7) and *aqp8a.1^−/−^* (*n* = 8) embryos in *Tg(fli1a:H2B-EGFP)* background (unpaired *t* test; *P* = 0.0255; two independent experiments). (**J**) AVC length in 48 hours postfertilization control (*n* = 7) and *aqp8a.1^−/−^* (*n* = 8) embryos in *Tg(fli1a:H2B-EGFP)* background, normalized to control (unpaired *t* test; P = 0.0025; two independent experiments). Control in (B) to (D) and (H) to (J): wild-type embryos/larvae in *Tg(fli1a:H2B-EGFP)* background; control in (E) and (F): wild-type embryos in *Tg(kdrl:NLS-EGFP);(fli1:Has.B4GALT1-mCherry)* background; control in (G): wild-type embryos. ns, not significant.

Cell hydraulics plays a pivotal role in morphogenesis, enabling dynamic changes that ultimately shape tissue architecture (*4*–*9*). Aquaporins, a class of membrane-spanning water channel proteins, primarily mediate the passive transport of water in response to osmotic gradients (*30*–*35*). In zebrafish, *aqp8a.1* expression is specifically enriched in the AV canal (AVC) and outflow tract (OFT) regions (*14*), where future valves develop. This spatial pattern coincides with the onset of AVC EdC volume reduction at 36 hours postfertilization, a critical morphogenetic step in heart valve formation (Fig. 1A) (*14*). To investigate the role of Aqp8a.1 in AV valve morphogenesis, we used a mutant zebrafish line carrying a deletion in *aqp8a.1* (*rk29*) that disrupts its water channel function (*36*). We found that *aqp8a.1* mutant larvae exhibited defective AV valve leaflets, characterized by a failure of the superior leaflet to elongate (Fig. 1B), highlighting the essential contribution of Aqp8a.1 to valve development. Given that changes in cell and nucleus volume are coupled during AVC morphogenesis in early zebrafish cardiogenesis (*14*), we measured EdC nucleus volume in mutant and control embryos as a proxy for cell volume. We observed that AVC EdC nucleus volume did not decrease but instead appeared significantly increased in *aqp8a.1^−/−^* embryos compared to controls at 48 hours postfertilization (Fig. 1, C and D), confirming that Aqp8a.1 is required for physiological EdC volume reduction. At 27 hours postfertilization, no significant difference in AVC EdC nucleus volume was detected between *aqp8a.1* mutants and controls (fig. S1, A and B), indicating that the phenotype is not yet evident at this early stage. However, at 54 hours postfertilization, the reduction in AVC EdC nucleus volume observed in control embryos remained absent in *aqp8a.1* mutants (fig. S1, C and D), demonstrating a persistent defect in volume regulation. To further assess the impact of Aqp8a.1 on cardiac morphogenesis, we examined cell polarity and F-actin remodeling, which are reminiscent of AVC formation (*14*). We analyzed nucleus-to-Golgi axis orientation using *Tg(kdrl:NLS-EGFP);(fli1:Has.B4GALT1-mCherry)*, in which both the nucleus and Golgi apparatus of EdCs are labeled. In *aqp8a.1* mutants, the proportion of ventricular EdCs with a nucleus-to-Golgi axis oriented toward the inflow tract was significantly reduced, whereas atrial cell orientation was unaffected (Fig. 1, E and F). In addition, we observed impaired F-actin remodeling in AVC EdCs of *aqp8a.1*^−/−^ embryos (Fig. 1G and fig. S1E). Notably, these mutants displayed defects in cardiac looping (Fig. 1, H and I) and a significant increase in AVC length (Fig. 1J), indicating broader morphogenetic disruptions across the heart. These findings demonstrate that Aqp8a.1 is required for coordinating cell polarity and cytoskeletal dynamics necessary for AV valve formation and heart looping.

**Fig. 2.**
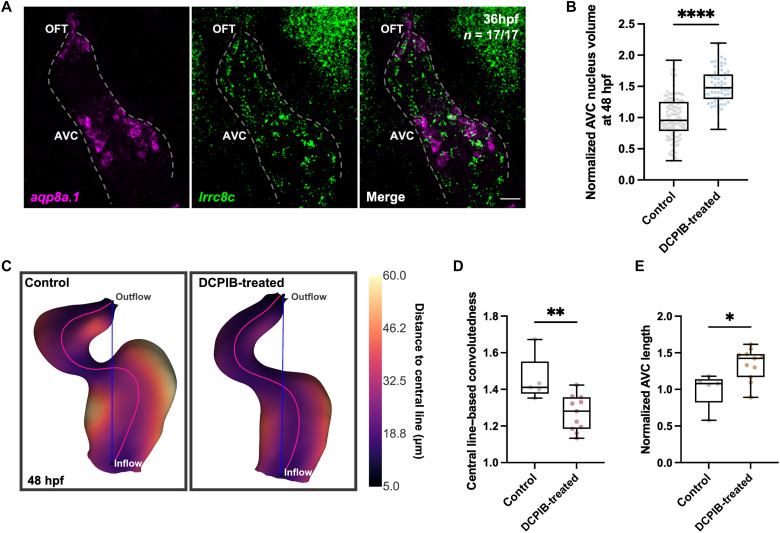
VRAC is a critical regulator of AVC EdC volume dynamics. (**A**) Maximum projections of RNAscope images showing the mRNA distribution of *aqp8a.1* and *lrrc8c* in the embryonic heart of wild-type zebrafish embryos (*n* = 17) at 36 hours postfertilization (three independent experiments). Dotted lines outline the endocardium. Scale bar, 20 μm. (**B**) Quantification of AVC nucleus volume in control (*n* = 110 nuclei/6 embryos) and DCPIB-treated (*n* = 59 nuclei/11 embryos) embryos in *Tg(kdrl:NLS-mCherry)* background at 48 hours postfertilization, normalized to mean control value (unpaired *t* test; *P* < 0.0001; one experiment, three independent clutches). (**C**) Representative 3D visualizations of control and DCPIB-treated hearts at 48 hours postfertilization. (**D**) Quantification of central line–based convolutedness in control (*n* = 5) and DCPIB-treated (*n* = 11) embryos in *Tg(kdrl:NLS-mCherry)* background at 48 hours postfertilization (Mann-Whitney test; *P* = 0.0087; one experiment, three independent clutches). (**E**) Quantification of AVC length in control (*n* = 5) and DCPIB-treated (*n* = 11) embryos in *Tg(kdrl:NLS-mCherry)* background at 48 hours postfertilization, normalized to mean control value (Mann-Whitney test; *P* = 0.0192; one experiment, three independent clutches). Control in (B) to (E): ethanol-treated embryos in *Tg(kdrl:NLS-mCherry)* background.

At the molecular level, water flux across the cell membrane is regulated by a coordinated set of aquaporins and ion channels, driven by osmotic forces (*37*–*40*). The VRAC plays a key role in cellular volume control in response to osmotic changes. SWELL1 (Lrrc8a) and Lrrc8c are functional components of the VRAC complex (*41*, *42*). In zebrafish, we found that *lrrc8aa*, the gene encoding SWELL1, is expressed in both the endocardium and myocardium of the developing heart (fig. S2A). In contrast, *lrrc8c* expression is primarily observed in the endocardium, where it is coexpressed with *aqp8a.1* in AVC EdCs (Fig. 2A and fig. S2, B and C), suggesting a potential interaction or coordinated function during valve development. Pharmacological inhibition of VRAC activity with the small molecule 4-[(2-Butyl-6,7-dichloro-2-cyclopentyl-2,3-dihydro-1-oxo-1*H*-inden-5-yl)oxy]butanoic acid (DCPIB) from 30 to 48 hours postfertilization resulted in significantly increased AVC EdC nucleus volume (Fig. 2B), defective cardiac looping (Fig. 2, C and D), an increase in AVC length (Fig. 2E), and altered heart rate (fig. S2D) compared to controls. These phenotypes closely resemble those observed in *aqp8a.1* mutants, indicating that VRAC-mediated ion flux is necessary for water efflux and EdC volume regulation. Together, our findings show that cell hydraulics mediated by VRAC and Aqp8a.1 is an essential driver of EdC volume reduction and AV valve morphogenesis, acting in coordination.

### Mechanical forces govern Aqp8a.1 plasma membrane localization

**Fig. 3.**
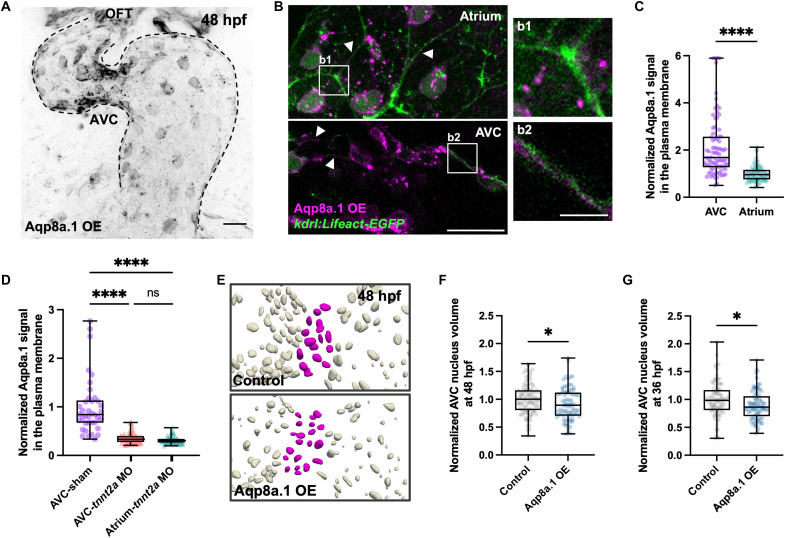
Mechanical forces control Aqp8a.1 plasma membrane localization, promoting AVC EdC volume reduction. (**A**) Confocal image of a 48 hours postfertilization Aqp8a.1 OE [*Tg(fli1ep:aqp8a.1-mCherry)*] heart. Dotted lines outline the endocardium. Scale bar, 20 μm. (**B**) Multiple *z*-plane projection image showing regions of the plasma membrane, marked by Lifeact, in atrial EdCs of a 48 hours postfertilization *Tg(fli1ep:aqp8a.1-mCherry);(kdrl:Lifeact-EGFP)* heart, and single *z*-plane image showing plasma membrane regions in AVC EdCs of a 48 hours postfertilization *Tg(fli1ep:aqp8a.1-mCherry);(kdrl:Lifeact-EGFP)* heart. White arrows indicate ROIs at the plasma membrane. Scale bars, 20 and 5 μm (insets). (**C**) Aqp8a.1 signal at the plasma membrane of AVC (*n* = 79 ROIs/9 embryos) and atrial (*n* = 85 ROIs/9 embryos) EdCs in 48 hours postfertilization *Tg(fli1ep:aqp8a.1-mCherry);(kdrl:Lifeact-EGFP)* embryos, normalized to mean AVC value (Mann-Whitney test; *P* < 0.0001; two independent experiments). (**D**) Aqp8a.1 signal at the plasma membrane of AVC and atrial EdCs in 48 hours postfertilization *Tg(fli1ep:aqp8a.1-mCherry);(kdrl:Lifeact-EGFP)* sham controls (*n* = 38 AVC ROIs/6 embryos) and *tnnt2a* morphants (*n* = 60 AVC ROIs and *n* = 60 atrial ROIs/9 embryos), normalized to mean AVC sham control value (Kruskal-Wallis test; both comparisons, *P* < 0.0001; one experiment, three independent clutches). (**E**) Representative images of segmented endocardial nuclei (gray) of 48 hours postfertilization control and Aqp8a.1 OE [*Tg(fli1ep:aqp8a.1-mEmerald)*] embryos in *Tg(kdrl:NLS-mCherry)* background, with AVC nuclei highlighted in magenta. (**F**) AVC nucleus volume in 48 hours postfertilization control (*n* = 98 nuclei/5 embryos) and Aqp8a.1 OE [*Tg(fli1ep:aqp8a.1-mEmerald)*] (*n* = 81 nuclei/4 embryos) embryos in *Tg(kdrl:NLS-mCherry)* background, normalized to control (unpaired *t* test; *P* = 0.0402; one experiment, two independent clutches). (**G**) AVC nucleus volume in 36 hours postfertilization control (*n* = 89 nuclei/6 embryos) and Aqp8a.1 OE [*Tg(fli1ep:aqp8a.1-mEmerald)*] (n = 85 nuclei/9 embryos) embryos in *Tg(kdrl:NLS-mCherry)* background, normalized to control (Mann-Whitney test; *P* = 0.0123; two independent experiments). Control in (E) to (G): wild-type embryos in *Tg(kdrl:NLS-mCherry)* background.

Having established the key role of Aqp8a.1 in EdC volume regulation, we set out to uncover the molecular mechanism by which it mediates this process. Aquaporin-mediated water flux is typically regulated by the insertion of aquaporins into the cell membrane to increase permeability and their removal to decrease it (*35*, *37*, *40*, *43*, *44*). To test this, we generated a transgenic zebrafish line for Aqp8a.1 overexpression [*Tg(fli1ep:aqp8a.1-mCherry)*], hereafter referred to as “Aqp8a.1 OE.” Aqp8a.1 was primarily localized to the plasma membrane and the perinuclear region of EdCs, with numerous protein aggregates also observed (Fig. 3A). Although Aqp8a.1 was expressed under the *fli1ep* promoter, which would typically lead to an even distribution of the protein across the endocardium, we observed a significant enrichment at the plasma membrane of AVC EdCs (Fig. 3, B and C, and fig. S3A). Given that wall shear stress is notably elevated at the AVC (*20*, *45*), we wondered whether mechanical forces could regulate the localization of Aqp8a.1. To this end, we analyzed Aqp8a.1 distribution across the endocardium in *cardiac troponin T type 2a* (*tnnt2a*) morphants, which lack heart contraction and, consequently, blood flow (*46*). In *tnnt2a* morphants, Aqp8a.1 levels at the plasma membrane of AVC EdCs were significantly reduced compared to those in sham controls (Fig. 3D), suggesting that mechanical forces promote Aqp8a.1 incorporation into the plasma membrane.

We observed a significant reduction in AVC EdC nucleus volume in embryos overexpressing Aqp8a.1 compared to controls at 48 hours postfertilization (Fig. 3, E and F), suggesting that Aqp8a.1 overexpression is sufficient to enhance EdC volume reduction. In addition, we found a similarly significant decrease at 36 hours postfertilization, comparable in magnitude to the reduction observed at 48 hours postfertilization (Fig. 3G), indicating that Aqp8a.1 overexpression accelerates EdC volume reduction. Together, these findings demonstrate that mechanical forces regulate Aqp8a.1 localization to the plasma membrane, promoting EdC volume reduction during early valve morphogenesis.

### CaM activity drives aquaporin localization dynamics

**Fig. 4.**
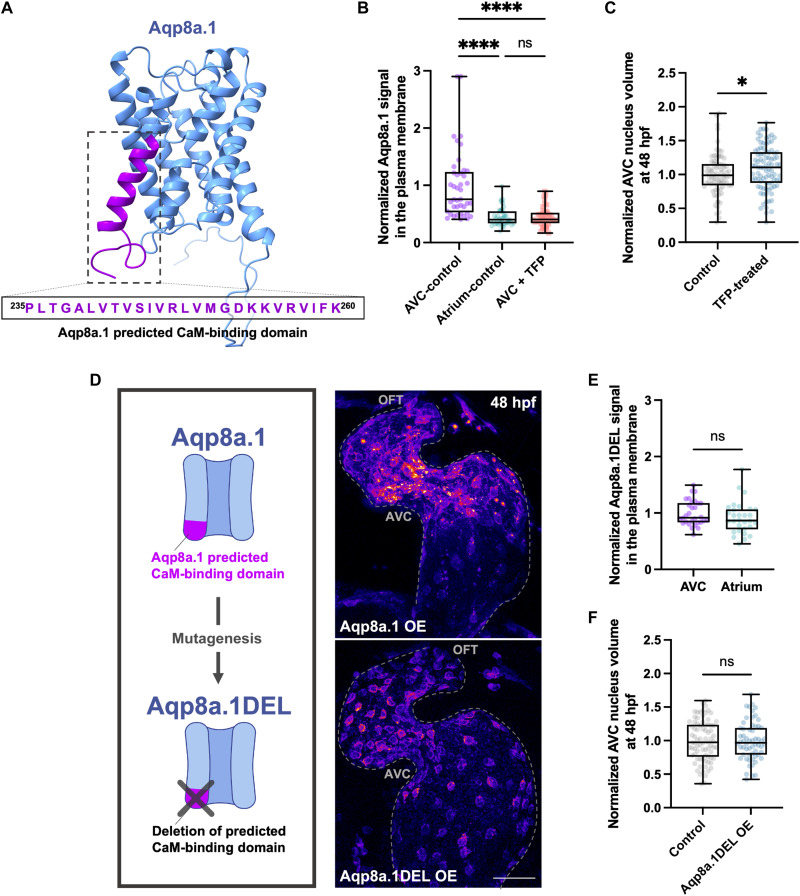
Aqp8a.1 localization is controlled by CaM activity. (**A**) Structural schematic of Aqp8a.1, predicted using AlphaFold (*72*), highlighting the CaM-binding site at its carboxyl terminus. (**B**) Quantification of Aqp8a.1 signal at the plasma membrane of AVC and atrial EdCs in *Tg(fli1ep:aqp8a.1-mCherry);(kdrl:Lifeact-EGFP)* control (*n* = 41 AVC ROIs and *n* = 40 atrial ROIs/4 embryos) and TFP-treated (*n* = 41 AVC ROIs/4 embryos) embryos at 48 hours postfertilization, normalized to mean AVC control value (Kruskal-Wallis test; both comparisons, *P* < 0.0001; one experiment, three independent clutches). (**C**) Quantification of AVC nucleus volume in control (*n* = 81 nuclei/5 embryos) and TFP-treated (*n* = 90 nuclei/6 embryos) embryos in *Tg(kdrl:NLS-mCherry)* background at 48 hours postfertilization, normalized to mean control value (Mann-Whitney test; *P* = 0.0361; one experiment, three independent clutches). (**D**) Schematic illustrating the site-directed mutagenesis strategy for Aqp8a.1 and representative confocal images of Aqp8a.1 OE and Aqp8a.1DEL OE zebrafish hearts at 48 hours postfertilization. Dotted lines outline the endocardium. Scale bar, 30 μm. (**E**) Quantification of Aqp8a.1DEL signal at the plasma membrane of AVC (*n* = 30 ROIs/6 embryos) and atrial (*n* = 30 ROIs/6 embryos) EdCs in Aqp8a.1DEL OE embryos at 48 hours postfertilization, normalized to mean AVC value (unpaired *t* test; ns; one experiment, three independent clutches). (**F**) Quantification of AVC nucleus volume in control (*n* = 79 nuclei/7 embryos) and Aqp8a.1DEL OE (*n* = 60 nuclei/5 embryos) embryos in *Tg(kdrl:NLS-mCherry)* background at 48 hours postfertilization, normalized to mean control value (unpaired *t* test; ns; two independent experiments). Control in (B) and (C): dH_2_O-treated embryos in *Tg(kdrl:NLS-mCherry)* background; control in (F): wild-type embryos in *Tg(kdrl:NLS-mCherry)* background. hpf, hours postfertilization.

Intracellular calcium levels are highest at the AVC cells, in response to the elevated mechanical forces in this region (*13*, *21*, *24*). Considering that the calcium-binding protein CaM can control the localization of aquaporins in response to intracellular calcium levels (*37*, *47*, *48*), we examined whether Aqp8a.1 localization is CaM dependent. Using the Calmodulin Target Database (*49*), which predicts CaM-binding sites within protein sequences, we identified a putative CaM-binding site at the carboxyl terminus of the protein, spanning the last residues 235 to 260 (Fig. 4A). When CaM was inhibited using the antagonist trifluoperazine dihydrochloride (TFP) in *Tg(fli1ep:aqp8a.1-mCherry);(kdrl:Lifeact-EGFP)* double transgenic embryos from 30 to 48 hours postfertilization, without affecting heart rate (fig. S3B), we observed decreased Aqp8a.1 levels at the plasma membrane of AVC EdCs, comparable to those observed in atrial EdCs (Fig. 4B and fig. S3C). In addition, TFP treatment led to a significant increase in AVC EdC nucleus volume (Fig. 4C), indicating that CaM activity controls the incorporation of Aqp8a.1 into the plasma membrane, and consequently EdC volume changes.

We next used a targeted site-directed mutagenesis approach to generate a mutant version of Aqp8a.1 lacking the 26-amino acid CaM-binding domain (residues 235 to 260), resulting in the transgenic line *Tg(fli1ep:aqp8a.1*^Δ*235*^*-mEmerald)*, hereafter referred to as “Aqp8a.1DEL OE” (Fig. 4D). In Aqp8a.1DEL OE embryos, the mutated protein was predominantly localized to the perinuclear region of EdCs, with reduced levels at the plasma membrane of AVC EdCs and almost no observable protein aggregates (Fig. 4, D and E, and fig. S3D). Moreover, overexpression of Aqp8a.1DEL did not result in a reduction in AVC EdC volume at either 36 or 48 hours postfertilization (Fig. 4F and fig. S3E), confirming that interaction with CaM is essential for proper Aqp8a.1 localization and its role in EdC volume regulation.

**Fig. 5.**
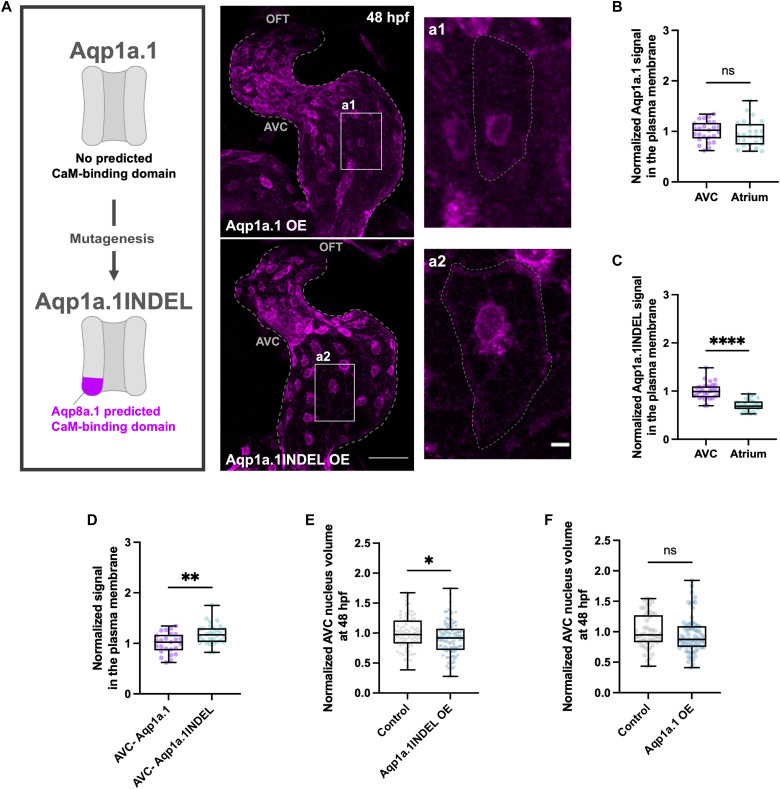
The CaM-binding motif is sufficient to determine aquaporin localization dynamics. (**A**) Schematic illustrating the site-directed mutagenesis strategy for Aqp1a.1 and representative confocal images of Aqp1a.1 OE and Aqp1a.1INDEL OE zebrafish hearts at 48 hours postfertilization. Dotted lines outline the endocardium (whole-heart images) and the plasma membrane boundaries of EdCs (insets). Scale bars, 30 and 5 μm (inset). (**B**) Quantification of Aqp1a.1 signal at the plasma membrane of AVC (*n* = 25 ROIs/5 embryos) and atrial (*n* = 25 ROIs/5 embryos) EdCs in Aqp1a.1 OE embryos at 48 hours postfertilization, normalized to mean AVC value (unpaired *t* test; ns; one experiment, three independent clutches). (**C**) Quantification of Aqp1a.1INDEL signal at the plasma membrane of AVC (*n* = 30 ROIs/6 embryos) and atrial (n = 30 ROIs/6 embryos) EdCs in Aqp1a.1INDEL OE embryos at 48 hours postfertilization, normalized to mean AVC value (unpaired *t* test; *P* < 0.0001; one experiment, three independent clutches). (**D**) Quantification of signal at the plasma membrane of AVC EdCs in Aqp1a.1 OE (*n* = 25 ROIs/5 embryos) and Aqp1a.1INDEL OE (*n* = 30 ROIs/6 embryos) embryos at 48 hours postfertilization, normalized to mean AVC Aqp1a.1 value (unpaired *t* test; *P* = 0.0016; one experiment, three independent clutches). (**E**) AVC nucleus volume in 48 hours postfertilization control (*n* = 76 nuclei/4 embryos) and Aqp1a.1INDEL OE (*n* = 110 nuclei/6 embryos) embryos in *Tg(kdrl:NLS-mCherry)* background, normalized to mean control value (unpaired *t* test; *P* = 0.0223; one experiment, two independent clutches). (**F**) AVC nucleus volume in 48 hours postfertilization control (*n* = 50 nuclei/6 embryos) and Aqp1a.1 OE (*n* = 68 nuclei/6 embryos) embryos in *Tg(kdrl:NLS-mCherry)* background, normalized to mean control value (Mann-Whitney test; ns; two independent experiments). Control in (E) and (F): wild-type embryos in *Tg(kdrl:NLS-mCherry)* background. The datasets shown in (D) are the same as those in (B) and (C). hpf, hours postfertilization.

We have previously shown that Aqp1a.1 is also expressed throughout the endocardium during the early stages of AV valve development in the zebrafish heart, with a subtle enrichment at the AVC (*14*). However, analysis of the Aqp1a.1 sequence using the Calmodulin Target Database revealed no predicted CaM-binding sites. This absence suggests that Aqp1a.1 may not be involved in AVC EdC volume regulation, as confirmed by the observation that *aqp8a.1;aqp1a.1* double mutants (*36*) exhibited the same phenotype as the *aqp8a.1* single mutants (fig. S3F), indicating a lack of additive function by Aqp1a.1 in this context. Nonetheless, Aqp1a.1 provided a valuable framework to directly test the importance of aquaporin-CaM interactions in this process. We reasoned that if CaM interaction is critical, Aqp1a.1 would mimic Aqp8a.1 shuttling in the cell membrane if the CaM-binding site were present. We thus generated a chimeric version of Aqp1a.1 by replacing its C-terminal 26 amino acids with the corresponding CaM-binding sequence of Aqp8a.1 (residues 235 to 260), resulting in a protein of identical length (Aqp1a.1INDEL) (Fig. 5A).

When Aqp1a.1 was overexpressed, it mainly localized to the plasma membrane and perinuclear region of EdCs (Fig. 5A), with an even distribution across the endocardium (Fig. 5B). In contrast, Aqp1a.1INDEL exhibited a distinct localization pattern, showing significant enrichment at the plasma membrane of AVC EdCs (Fig. 5, A and C, and fig. S3G) and higher levels compared to wild-type Aqp1a.1 (Fig. 5D), a pattern reminiscent of the wild-type Aqp8a.1 distribution. Notably, overexpression of Aqp1a.1INDEL resulted in a reduction in AVC EdC volume at 48 hours postfertilization (Fig. 5E), whereas Aqp1a.1 had no effect (Fig. 5F). These findings show that insertion of the CaM-binding motif is sufficient to confer the localization pattern observed with Aqp8a.1 and to directly control EdC volume reduction.

Overall, our results reveal that CaM-dependent regulation of aquaporin localization is a key mechanism controlling EdC volume, with the CaM-binding motif acting as a critical determinant of Aqp8a.1 incorporation in the cell membrane to activate cell volume regulation during early cardiac morphogenesis.

### Piezo1 acts through a dual mechanism to regulate EdC volume

**Fig. 6.**
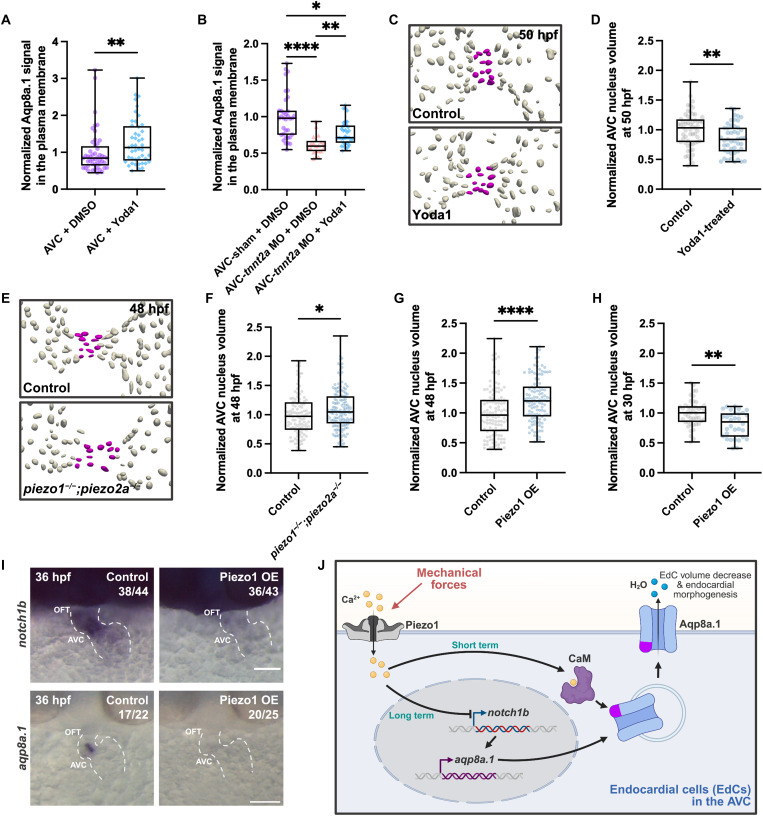
Piezo1 regulates AVC EdC volume by controlling Aqp8a.1 localization and expression. (**A**) Aqp8a.1 signal at the plasma membrane of AVC EdCs in 48 hours postfertilization *Tg(fli1ep:aqp8a.1-mCherry);(kdrl:Lifeact-EGFP)* DMSO-treated (*n* = 48 ROIs/4 embryos) and Yoda1-treated (*n* = 45 ROIs/5 embryos) embryos, normalized to mean AVC + dimethyl sulfoxide (DMSO; Mann-Whitney test; *P* = 0.0076; one experiment, three independent clutches). (**B**) Aqp8a.1 signal in 48 hours postfertilization *Tg(fli1ep:aqp8a.1-mCherry);(kdrl:Lifeact-EGFP)* sham + DMSO (*n* = 35 ROIs/5 embryos), *tnnt2a* morphants + DMSO (*n* = 21 ROIs/3 embryos), and *tnnt2a* morphants + Yoda1 (*n* = 35 ROIs/5 embryos), normalized to mean AVC-sham + DMSO (Kruskal-Wallis test; *P* < 0.0001, 0.0047, and 0.0115; one experiment, three independent clutches). (**C**) Segmented endocardial nuclei of 48 hours postfertilization control and Yoda1-treated embryos, with AVC nuclei in magenta. (**D**) AVC nucleus volume in 50 hours postfertilization *Tg(kdrl:NLS-mCherry)* control (*n* = 58 nuclei/3 embryos) and Yoda1-treated (*n* = 47 nuclei/3 embryos) embryos, normalized to control (unpaired *t* test; *P* = 0.0054; one experiment, two independent clutches). (**E**) Segmented endocardial nuclei of 48 hours postfertilization control and *piezo1^−/−^;piezo2a^−/−^* embryos, with AVC nuclei in magenta. (**F**) AVC nucleus volume in 48 hours postfertilization control (*n* = 108 nuclei/8 embryos) and *piezo1^−/−^;piezo2a^−/−^* (*n* = 146 nuclei/12 embryos) embryos, normalized to control (Mann-Whitney test; *P* = 0.0440; two independent experiments). (**G**) AVC nucleus volume in 48 hours postfertilization control (*n* = 120 nuclei/11 embryos) and Piezo1 OE (*n* = 115 nuclei/9 embryos) embryos, normalized to control (Mann-Whitney test; *P* < 0.0001; two independent experiments). (**H**) AVC nucleus volume in 30 hours postfertilization *Tg(kdrl:NLS-mCherry)* control (*n* = 45 nuclei/8 embryos) and Piezo1 OE (*n* = 32 nuclei/7 embryos) embryos, normalized to control (Mann-Whitney test; *P* = 0.0010; two independent experiments). (**I**) In situ hybridization of *notch1b* and *aqp8a.1* in 36 hours postfertilization control and Piezo1 OE embryos (four independent experiments). Dotted lines indicate heart shape. Scale bars, 20 μm. (**J**) Model for AVC EdC volume regulation. Created in BioRender. Vagena-Pantoula, C. (2025) https://BioRender.com/6zfn1cn. Control in (C) and (D): DMSO-treated embryos; control in (E) and (F): *piezo1^+/−^;piezo2a^+/+^*, *piezo1^+/+^;piezo2a^+/+^* embryos; control in (G) to (I): *Tg(hsp70l:GenEPi)* nonheat-shocked siblings. hpf, hours postfertilization.

Considering the mechanosensitive regulation of Aqp8a.1 localization and EdC volume changes, we investigated whether mechanically activated ion channels contribute to these mechanisms. As Piezo ion channels are essential for the formation of functional AV and OFT valves (*22*, *24*, *28*), and Piezo1 has been implicated in regulating cell volume homeostasis in red blood cells in zebrafish (*29*) and mouse models (*50*), we focused on their potential role in EdC volume regulation. We first activated Piezo1 pharmacologically using Yoda1 from 30 to 48 hours postfertilization in *Tg(fli1ep:aqp8a.1-mCherry);(kdrl:Lifeact-EGFP)* embryos. Despite a reduction in heart rate (fig. S4A), Yoda1 treatment enhanced Aqp8a.1 localization to the plasma membrane in both AVC and atrial EdCs (Fig. 6A and fig. S4B), indicating that Piezo1 activation promotes its plasma membrane incorporation. Notably, a 2-hour Yoda1 treatment in *tnnt2a* morphants partially restored Aqp8a.1 plasma membrane localization in AVC EdCs (Fig. 6B), suggesting that Piezo1 activity can compensate, at least in part, for the absence of contractile force and blood flow. Extended Yoda1 treatment in *tnnt2a* morphants led to lethality, preventing evaluation of prolonged effects.

To test whether Piezo1 activity regulates EdC volume, and given that volume changes are expected to occur rapidly due to protein-level regulation, we performed a brief 2-hour treatment with Yoda1 from 48 to 50 hours postfertilization. Yoda1-treated embryos exhibited significantly reduced AVC EdC nucleus volume compared to controls (Fig. 6, C and D), despite a decrease in heart rate (fig. S4C), suggesting that Piezo1 activation is sufficient to drive EdC shrinkage. Conversely, *piezo1;piezo2a* double mutant embryos (*24*) showed a significant increase in AVC EdC nucleus volume relative to controls (Fig. 6, E and F), indicating that Piezo function is required for EdC volume regulation. To further examine the effects of Piezo1 activity, we used a transgenic line *Tg(hsp70l:GenEPi)* (*51*), referred to as “Piezo1 OE,” which allows for conditional overexpression of Piezo1 upon heat shock. To validate that Piezo1 overexpression alone using GenEPi (*51*) leads to increased intracellular calcium levels, we first tested its effect in human embryonic kidney (HEK) 293T cells and observed a significant elevation, comparable to the shear stress–induced response in nonoverexpressing cells (fig. S5, A and B). Sustained Piezo1 overexpression for ~20 hours resulted in EdC swelling at 48 hours postfertilization (Fig. 6G) without changes in heart rate (*51*). In contrast, short-term Piezo1 overexpression for ~1 to 3 hours led to reduced EdC nucleus volume at 30 hours postfertilization (Fig. 6H), despite a decreased heart rate (*51*). Notably, Yoda1 treatment for 3 and 20 hours recapitulated these effects at 30 and 48 hours postfertilization (fig. S5, C and D), respectively, further supporting our findings. These results highlight the importance of tightly regulated Piezo1 activity in maintaining EdC volume homeostasis, where transient activation promotes cell shrinkage, while sustained overactivation leads to cell swelling.

To investigate the mechanism underlying Piezo1-induced EdC swelling, we hypothesized that sustained Piezo1 activity controls volume homeostasis through transcriptional regulation. In situ hybridization at 36 hours postfertilization revealed that prolonged Piezo1 overexpression, initiated at approximately 28 hours postfertilization, led to the down-regulation of both *notch1b* and *aqp8a.1* expression (Fig. 6I). Given prior evidence linking Notch signaling to aquaporin regulation in other systems (*52*), and since suppression of Notch signaling leads to increased AVC EdC volume in our model (*14*), we tested whether Notch signaling regulates *aqp8a.1* transcription. Notch pathway blockade using N-[N-(3,5-Difluorophenacetyl)-L-alanyl]-S-phenylglycine t-butyl ester (DAPT) from 30 to 36 hours postfertilization significantly reduced *aqp8a.1* expression (fig. S6, A and B), without affecting heart rate (fig. S6C), supporting a model in which Notch1b acts upstream of *aqp8a.1* in EdCs.

Together, these findings reveal a dual mechanism by which Piezo1 controls EdC volume: in the short-term, by promoting Aqp8a.1 plasma membrane localization, and in the long-term, through Notch1b-dependent transcriptional repression of *aqp8a.1* (Fig. 6J). This dual mode of regulation enables tight homeostatic control of EdC size during cardiac morphogenesis.

## DISCUSSION

Our findings uncover a mechanosensitive, hydraulics-based mechanism that governs cardiac valve morphogenesis by regulating EdC volume through controlled water flux. We propose that mechanotransduction actively drives morphogenesis by modulating cell volume, rather than acting solely through transcriptional programs. The identification of Piezo1 as a dual regulator of Aqp8a.1, controlling both its plasma membrane localization via CaM and its transcription via Notch1b, reveals a sophisticated interplay between mechanical stimuli, ion flux, gene expression, and water transport in cardiac development.

By placing cell hydraulics at the core of cardiac tissue morphogenesis, our study broadens the conceptual framework for how mechanical forces sculpt organ architecture. While extensive research has elucidated roles for cell migration (*53*, *54*), contractility (*14*, *55*, *56*), and polarity (*14*, *57*, *58*) in shaping tissues, remarkably little is known about how cell volume is regulated during development, and how volume changes contribute to morphogenesis. Our findings begin to fill this gap, positioning volume control as a key and active determinant of developmental outcomes. The finding that aquaporin-mediated water efflux is not merely permissive but actively required for EdC volume reduction and valve formation suggests that modulation of cell size itself functions as a morphogenetic signal. The functional coupling of Aqp8a.1 and VRAC further underscores the importance of coordinating ionic and osmotic responses during development, drawing parallels with volume regulatory mechanisms observed in diverse systems (*32*, *36*, *37*, *59*). These findings point to hydraulics-based morphogenesis as a potentially widespread mechanism in organ development, highlighting the need for further investigation.

Moreover, the identification of a Piezo1-CaM-Aqp8a.1 axis as a tunable effector of cell size suggests a broader principle of mechanical homeostasis during morphogenesis. Transient Piezo1 activation promotes cell shrinkage via Aqp8a.1 membrane insertion, while sustained activity initiates a feedback loop suppressing *aqp8a.1* transcription via Notch1b signaling, enabling precise control over EdC volume. This biphasic regulation reflects a system optimized for both responsiveness and stability, revealing how developmental programs balance mechanical sensitivity with morphogenetic fidelity.

Our study also raises broader questions about how the spatial organization of hydraulic signaling contributes to tissue morphogenesis in vivo. While aquaporins have been widely implicated in fluid transport and homeostasis (*32*, *36*, *37*, *39*, *40*), their regulation by mechanical forces adds an additional layer to their functional repertoire. The localized enrichment of Aqp8a.1 at the AVC, a site of heightened shear stress (*13*, *24*), positions aquaporins as downstream effectors of hemodynamic patterning. Whether similar spatial regulation governs other morphogenetic contexts, such as epithelial folding, angiogenesis, neural tube closure, or placental villi formation, remains to be determined.

These findings may have potential implications beyond development. The core components of this pathway, Piezo channels, aquaporins, and Notch signaling, remain expressed in adult tissues and are implicated in a range of diseases, including cardiomyopathies, edema, and cancer (*30*, *37*, *50*, *60*–*64*). Understanding how mechanical forces intersect with cell volume regulation in pathological contexts may open therapeutic avenues targeting aquaporin trafficking or Piezo activity.

In summary, we propose that mechanical forces drive morphogenesis not only by regulating gene expression but also through physical modulation of cell volume via controlled water flux. This hydraulics-based mechanism, governed by a Piezo1-CaM-Aqp8a.1 circuit, provides a dynamic strategy for translating mechanical inputs into morphogenetic outcomes. By establishing mechanohydraulics as a central determinant of cardiac valve development, our study lays the groundwork for a broader reevaluation of mechanical forces as active drivers of morphogenesis across vertebrate development.

## MATERIALS AND METHODS

### Zebrafish husbandry

Animal experiments conducted in the UK adhered to the European directive 2010/63/EU and Home Office guidelines under the project license PP6020928. Animal experiments conducted in Japan were approved by the Institutional Animal Care and Use Committee at RIKEN Kobe Branch. Embryos were raised at 28.5°C and treated with 0.003% 1-phenyl-2-thiourea (Sigma-Aldrich, P7629) after 24 hours postfertilization to prevent pigment formation. We used the AB strain as wild type. The following published zebrafish lines were used in this study: *aqp1a.1^rk28^* (*36*), *aqp8a.1^rk29^* (*36*), *piezo1^bns340^* (*24*), *piezo2a^bns367^* (*24*), *Tg(hsp70l:GenEPi)* (*51*), *Tg(kdrl:NLS-mCherry)^is4^* (*65*), *Tg(kdrl:NLS-EGFP)^ubs1^;(fli1:Has.B4GALT1-mCherry)^bns9^* (*66*), *Tg(kdrl:Lifeact-EGFP)^s982^* (*67*), and *Tg(fli1a:H2B-EGFP)^ncv69^* [renamed in ZFIN as *Tg(fli1:zgc:114046-EGFP)*] (*68*). Newly generated transgenic lines included the following: *Tg(fli1ep:aqp8a.1-mCherry)*, *Tg(fli1ep:aqp8a.1-mEmerald)*, *Tg(fli1ep:aqp1a.1-mEmerald)*, *Tg(fli1ep:aqp8a.1*^Δ*235*^*-mEmerald)*, and *Tg(fli1ep:aqp1a.1^Indel^-mEmerald)*. Control embryos were stage-matched wild-type or nontransgenic siblings, depending on the experiment. Genotyping of mutant and wild-type alleles was performed as previously described (*24*, *36*). Polymerase chain reaction amplifications were carried out using KAPA2G Fast Ready Mix (Sigma-Aldrich, KK5101).

### Mutagenesis

Site-directed mutagenesis was performed using the Q5 Site-Directed Mutagenesis Kit (New England Biolabs, NEB, E0554S) according to the manufacturer’s guidelines. Primers for site-directed mutagenesis were designed using NEBaseChanger (https://nebasechanger.neb.com/) and are provided in table S1. Sequencing of clones was conducted by Eurofins Genomics (https://eurofinsgenomics.eu/). Plasmid DNA extraction was performed using the Qiagen Plasmid Midi Kit (QIAGEN, 12143).

### *tnnt2a* morpholino injections

To inhibit cardiac contraction, 5.8 ng of the *tnnt2a* morpholino (5′-CATGTTTGCTCTGATCTGACACGCA-3′) (GeneTools) (*46*) was injected at the one-cell stage in a volume of 2.3 nl. Sham controls were injected with an equal volume of 5 mM Hepes buffer containing 0.05% phenol red, but without morpholino.

### Confocal imaging

For fluorescent imaging of the live heart, dechorionated zebrafish embryos were anesthetized in 0.02% tricaine (Sigma-Aldrich, A5040) or treated with 50 mM 2,3-butanedione monoxime (BDM; Sigma-Aldrich, B0753) to inhibit heart contraction. Anesthetized embryos were mounted on a glass-bottom petri dish (MatTek, P35G-0-14-C) in 1.2% low-melting point agarose (Invitrogen, 16520050) in E3 medium supplemented with the same concentration of tricaine or BDM as the medium.

Imaging at early developmental stages (27, 30, and 36 hours postfertilization) was performed on deyolked embryos fixed at the stage of interest for 2 to 3 hours at room temperature (RT) (or for 10 hours at 4°C in the case of 36 hours postfertilization) in 4% (w/v) paraformaldehyde (Alfa Aesar, A11313) in phosphate-buffered saline (PBS) supplemented with 4% (w/v) sucrose (Alfa Aesar, J64270) and 120 μM CaCl_2_ (Sigma-Aldrich, C1016) to facilitate deyolking. After fixation, embryos were washed in PBS containing 0.1% Tween 20 (PBST), deyolked, and mounted on glass-bottom petri dishes in 1.2% low-melting point agarose prepared in PBS.

Confocal imaging was performed on a Leica SP8 confocal laser scanning microscope equipped with a white light laser (WLL; Leica) using either an HC PL APO CS2 20×/0.75 dry objective or an HC PL APO CS2 63×/1.2 water-immersion objective. Imaging was also conducted on a Leica STELLARIS 8 confocal laser scanning microscope equipped with a WLL and an HC PL APO CS2 20×/0.75 dry objective. In addition, an inverted Olympus IX83/Yokogawa CSU-W1 spinning disk confocal microscope, equipped with a Zyla 4.2 complementary metal-oxide semiconductor camera (Andor) and an Olympus UPLSAPO 30×/1.05 silicone oil-immersion objective, was used.

### Immunofluorescence

Immunofluorescence experiments were performed on deyolked embryos. Zebrafish embryos were fixed at the stage of interest for 2 to 3 hours at RT or overnight at 4°C in 4% (w/v) paraformaldehyde (Alfa Aesar, A11313) in PBS supplemented with 4% (w/v) sucrose (Alfa Aesar, J64270) and 120 μM CaCl_2_ (Sigma-Aldrich, C1016) to facilitate deyolking. Following fixation, embryos were washed in PBST. For phalloidin (1:400; Alexa Fluor 488, Life Technologies, A12379) and anti-Fli1 staining, embryos were permeabilized with 1× PBS containing 0.5% Triton overnight at 4°C and subsequently blocked in PBS containing 0.1% Triton X-100, 5% bovine serum albumin (BSA) (SEQENS IVD, 1005-70), and 10% normal goat serum (NGS) (Coger, VS-1000) overnight at 4°C. Antibodies were used as follows: rabbit anti-Fli1 (1:100; Abcam, ab133485) and goat anti-rabbit Alexa Fluor 647 secondary antibody (1:500; Life Technologies, A-21235).

### Image analysis

#### Classification of cell polarity

Imaging was performed on deyolked embryos fixed at the stage of interest for 2 to 3 hours at RT in 4% (w/v) paraformaldehyde (Alfa Aesar, A11313) in PBS supplemented with 4% (w/v) sucrose (Alfa Aesar, J64270) and 120 μM CaCl_2_ (Sigma-Aldrich, C1016) to facilitate deyolking. After fixation, embryos were washed in PBST, deyolked, and mounted on glass-bottom petri dishes in 1.2% low-melting point agarose prepared in PBS.

Manual labeling of individual nuclei and Golgi apparatus was performed using the “cell counter” tool in FiJi/ImageJ (*69*). Cell polarity was classified visually based on the position of the Golgi apparatus relative to the cell nucleus: polarized toward the outflow, polarized toward the inflow, or no clear polarization.

#### Quantification of Aqp signal at the plasma membrane

In *Tg(fli1ep:aqp8a.1-mCherry);(kdrl:Lifeact-EGFP)* double transgenic embryos, 7 to 12 lines were drawn perpendicular to the plasma membrane in single *z* planes, based on the cortical actin signal (Lifeact channel), using FiJi/ImageJ (*69*). We used Lifeact as a proxy for the plasma membrane based on its strong cortical actin localization. Given the resolution limits of confocal microscopy, the cortical Lifeact signal closely overlaps with the plasma membrane, which allows us to use it as a spatial reference for membrane-associated dynamics. The same lines were then applied to the corresponding Aqp channel to measure maximum fluorescence intensity, which was subsequently used for statistical analysis.

For *Tg(fli1ep:aqp1a.1-mEmerald)*, *Tg(fli1ep:aqp8a.1*^Δ*235*^*-mEmerald)*, *Tg(fli1ep:aqp1a.1^Indel^-mEmerald)*, phalloidin staining was performed on deyolked embryos. Quantification was carried out using the same approach as described above: Five lines were drawn perpendicular to the plasma membrane in single *z* planes, guided by the phalloidin signal. Images were acquired on a Leica SP8 confocal laser scanning microscope equipped with a WLL (Leica) using an HC PL APO CS2 63×/1.2 water-immersion objective with 2.0× zoom, enabling observation of Aqp localization changes at the cellular level.

#### Quantification of phalloidin signal

For phalloidin-labeled immunofluorescence images, 5 to 10 EdCs in the AVC region were analyzed per embryo. In each cell, 7 to10 lines were drawn perpendicular to the plasma membrane on single *z* planes using FiJi/ImageJ (*69*), and the maximum fluorescence intensity along each line was measured for statistical analysis.

#### Nuclei segmentation

Nucleus volume analysis was conducted using imaging data from zebrafish embryos in either *Tg(kdrl:NLS-mCherry)^is4^* (*65*) or *Tg(fli1a:H2B-EGFP)^ncv69^* [renamed in ZFIN as *Tg(fli1:zgc:114046-EGFP)*] (*68*) background, when possible. In cases where these transgenic lines could not be used, endothelial and endocardial nuclei were visualized using anti-Fli1 immunostaining.

Nuclei segmentation was performed automatically using the deep learning model NISNet3D, trained and validated on a dataset of 57 zebrafish hearts, including both fixed and BDM-arrested samples. Ground truth labels for training were generated using an internally developed semiautomatic segmentation tool, designed for efficient three-dimensional (3D) blob instance labeling. This tool uses image processing filters, such as a multiscale Laplacian of Gaussian, combined with user input to create local 3D nuclei instances. This allowed generation of fully segmented 3D images in under 30 min per image, depending on complexity.

An iterative approach was used to expand the dataset using StarDist3D: Initial segmentations were generated and manually corrected to augment the training dataset. The first 10 images were segmented via the interface, while the final dataset comprised 67 images, with 10 images reserved as a testing set. StarDist3D was trained using a patch size of 96 × 96 × 96, batch size of 2, 100 steps per epoch for 500 epochs, 96 rays, and a UNet depth of 3. Remaining parameters followed the defaults of the public implementation (https://github.com/stardist/stardist).

Once the full dataset was curated, segmentation was performed using NISNet3D (https://gitlab.com/viper-purdue/nisnet-release) with a depth-3 ResAttentionUNet architecture (16 hidden layers at the first stage). Training was conducted with a patch size of 128 × 128 × 128 voxels, batch size of 4, over 500 epochs using the Adam optimizer with an initial learning rate of 0.001. Morphological postprocessing was disabled. Instead, validation samples were used to tune the remaining postprocessing parameters: Binning was set to 1, nonmaximum suppression distance to 3, ctr_threshold to 1, h_val to 0.5, k_val to 1, and vector decoding was enabled.

Nuclei were manually classified, with those located in the innermost region of the AVC assigned to the AVC class. Nucleus volume was computed using custom Python scripts applied to the labeled images.

#### Endocardial morphology analysis

Endocardial morphology analysis was conducted using live imaging data from zebrafish embryos in either *Tg(kdrl:NLS-mCherry)^is4^* (*65*) or *Tg(fli1a:H2B-EGFP)^ncv69^* [renamed in ZFIN as *Tg(fli1:zgc:114046-EGFP)*] (*68*) background. Automated segmentation of the endocardial lumen was achieved using a 3D UNet model trained on 213 labeled images. The training dataset was generated through an iterative process: Initial manual segmentations were used to train a model, which was then used to segment new images, later manually corrected to improve quality.

This segmentation task identified five anatomical compartments: pre-atrium, atrium, AVC, ventricle, and postventricle, using a region-based approach. To ensure consistency across samples, all images were rescaled to an isotropic resolution of 2 μm per voxel edge, enabling the model to capture broad contextual cues. The UNet used had a depth of 5, with 32 initial filters, deep supervision, and a patch size of 96 × 144 × 144. Training was carried out over 1000 epochs (250 steps per epoch), using a batch size of 2 and the AdamW optimizer with an initial learning rate of 0.001. A combined Dice and Cross-Entropy loss function was used.

To assess AVC geometry, the interfaces between the atrium and AVC and between the AVC and ventricle were identified. The centroids of these interfaces were then computed and connected to obtain the AVC length. Similarly, the centroids at the pre-atrium/atrium and ventricle/postventricle boundaries were located, allowing the definition of the “inflow” and “outflow” points. The straight-line distance between these points was compared to the length of the endocardial central line, and the ratio between these two lengths was used to define the looping ratio.

#### Chromogenic in situ hybridization

Whole-mount chromogenic in situ hybridization (ISH) was conducted following established protocols (*70*). The in situ probe for *aqp8a.1* was synthesized as previously described (*14*). The ISH samples were imaged using a Leica M165 C stereoscope fitted with Leica IC80 HD camera.

#### RNAscope ISH

RNAscope experiments were performed on deyolked embryos fixed overnight at 4°C in 4% (w/v) paraformaldehyde (Alfa Aesar, A11313) in PBS supplemented with 4% (w/v) sucrose (Alfa Aesar, J64270) and 120 μM CaCl_2_ (Sigma-Aldrich, C1016) to facilitate deyolking. RNAscope ISH was performed using the RNAscope Fluorescent Multiplex Reagent kit v2 (Advanced Cell Diagnostics, 323110), according to the manufacturer’s standard protocol. For quantification, the maximum intensity of the AVC RNAscope signal in the projection channel was measured and used for statistical analysis.

### Pharmacological treatments

#### DCPIB treatment

Zebrafish embryos were treated with 3 μM DCPIB (Tocris, 1540), diluted in ethanol, from 30 to 48 hours postfertilization, and kept at 28.5°C. Control embryos were treated with ethanol at the same final concentration and for the same duration.

#### Yoda1 treatment

Zebrafish embryos were treated with 25 μM Yoda1 (Sigma-Aldrich, SML1558), diluted in dimethyl sulfoxide (DMSO), from 27 to 30 hours postfertilization, 30 to 48 hours postfertilization, or 48 to 50 hours postfertilization, and kept at 28.5°C. Control embryos were treated with DMSO at the same final concentration and for the same duration.

#### DAPT treatment

Zebrafish embryos were treated with 100 μM DAPT (Sigma-Aldrich, D5942), diluted in DMSO, from 30 to 36 hours postfertilization, and kept at 28.5°C. Control embryos were treated with DMSO at the same final concentration and for the same duration.

#### TFP treatment

Zebrafish embryos were treated with 100 μg/ml TFP (Sigma-Aldrich, T8516), diluted in dH_2_O, from 30 to 48 hours postfertilization, and kept at 28.5°C. Control embryos were treated with dH_2_O at the same final concentration and for the same duration.

#### Heat shock of zebrafish embryos

Piezo1 overexpression in *Tg(hsp70l:GenEPi)* (*51*) was induced following a 1-hour heat shock at 37.5°C. For embryos used at 30 and 36 hours postfertilization, a heat shock was conducted at 24 hours postfertilization, as the signal typically develops after 3 to 5 hours (*51*). For embryos used at 48 hours postfertilization, a subsequent heat shock was performed at 34 hours postfertilization. Nonheat-shocked siblings were used as controls. The GenEPi system has previously been validated for the inducible expression of functional human Piezo1 that preserves physiological relevance across in vitro and in vivo contexts (*51*).

#### Cell culture and transfections

HEK293T doxycycline-inducible XLGenEPi stable cells, previously developed (*51*), were cultured at 37°C in 5% CO_2_ in high-glucose Dulbecco’s modified Eagle’s medium with GlutaMAX (Thermo Fisher Scientific, 10569010), supplemented with 10% fetal bovine serum (Thermo Fisher Scientific, A5256801) and 1× penicillin-streptomycin solution (Thermo Fisher Scientific, 15140122). Cells were routinely tested for mycoplasma contamination using a Mycoplasma detection kit (LuBioScience GmBH, B39032) and confirmed to be mycoplasma negative.

Plasmid DNA for transfections was isolated from a 30-ml LB culture (Thermo Fisher Scientific, 12780029) containing the appropriate antibiotics, using the Qiagen Plasmid Midi Kit (QIAGEN, 12143). DNA concentration was measured with a NanoDrop OneC Spectrophotometer (Thermo Fisher Scientific).

#### FLIM experiments

For fluorescence lifetime imaging microscopy (FLIM) experiments, HEK293T doxycycline-inducible XLGenEPi stable cells (*51*) were transfected with the pPB-3xnls-Tq-Ca-FLITS plasmid (*71*) (a gift from D. Gadella; Addgene plasmid #145030; http://n2t.net/addgene:145030; RRID:Addgene_145030) using Lipofectamine 3000 (Thermo Fisher Scientific, L3000001), following the manufacturer’s instructions.

Two days posttransfection, cells were washed, trypsinized with TrypLE Express (Thermo Fisher Scientific, 12604013), resuspended in full medium, and centrifuged at 1000 rpm for 5 min. The cell pellet was then resuspended and seeded onto 0.1% gelatin-coated, 20-mm-wide glass-bottom confocal dishes (VWR, 734-2906) at a density of 200,000 cells per well.

Six to 8 hours postseeding, the medium was replaced with full medium containing 500 ng/ml doxycycline (Merck, D9891) to induce GenEPi expression. After 16 hours, the medium was exchanged again for normal full medium before imaging.

Live-cell images were acquired using a Leica STELLARIS 8 confocal laser scanning microscope equipped with a WLL (Leica) and an HC PL APO CS2 20×/0.75 dry objective. Images were captured in photon-counting mode with excitation at 440 nm for Tq-Ca-FLITS (emission detection range: 445 to 495 nm) and at 503 nm for GenEPi (emission detection range: 507 to 607 nm). Lifetime imaging for Tq-Ca-FLITS was recorded at RT with no line averaging and five frame repetitions using LAS X FLIM software (Leica).

For the stimulation condition, individual glass-bottom dishes were incubated at RT in a benchtop orbital incubator at ~125 rpm for 15 min before direct imaging. Piezo1 agonism was induced by treating cells with 10 μM Yoda1 (Sigma-Aldrich, SML1558).

Regions of interest (individual nuclei) were selected using LAS X FLIM software to extract the average fluorescence lifetime for each condition. Only nuclei with appropriate average intensity were selected to avoid influence of background fluorescence (>100). The data were then plotted and analyzed using GraphPad Prism (v9).

### Statistical analysis

Statistical analyses were performed using GraphPad Prism (v9). The specific statistical tests applied, along with the numbers of analyzed nuclei, regions of interest (ROIs), embryos, and independent experiments, are indicated in the corresponding figure legends. Experiments performed once used embryos from at least two independent clutches; for repeated experiments, the number of independent experiments is indicated, each using embryos from at least two clutches. For cell-based assays, two independent biological replicates refer to two independent transfections performed on cells of different passages on the same day. *P* values are represented as follows: *P < 0.05, **P < 0.01, ***P < 0.001, and ****P < 0.0001. Box plots were generated using GraphPad Prism (v9), with horizontal lines representing the mean and whiskers indicating the minimum and maximum values. Sample sizes were not predetermined by statistical methods.
