# Supplementary material for: Piezo1-mediated mechanohydraulic control of cell volume drives cardiac morphogenesis
Source: Sci Adv. 2026 Apr 22;12(17):eaea7025. doi: 10.1126/sciadv.aea7025 (PMC13101866; doi:10.1126/sciadv.aea7025)
Supplement: Supplementary file 1 — Figs. S1 to S6 Table S1 [file sciadv.aea7025_sm.pdf]

Science Advances
AAAS

Supplementary Materials for

Piezo1-mediated mechanohydraulic control of cell volume drives cardiac morphogenesis

Christina Vagena-Pantoula *et al.*

Corresponding author: Julien Vermot, j.vermot@imperial.ac.uk

Sci. Adv. **12**, eaea7025 (2026)
DOI: 10.1126/sciadv.aea7025

This PDF file includes:

Figs. S1 to S6
Table S1

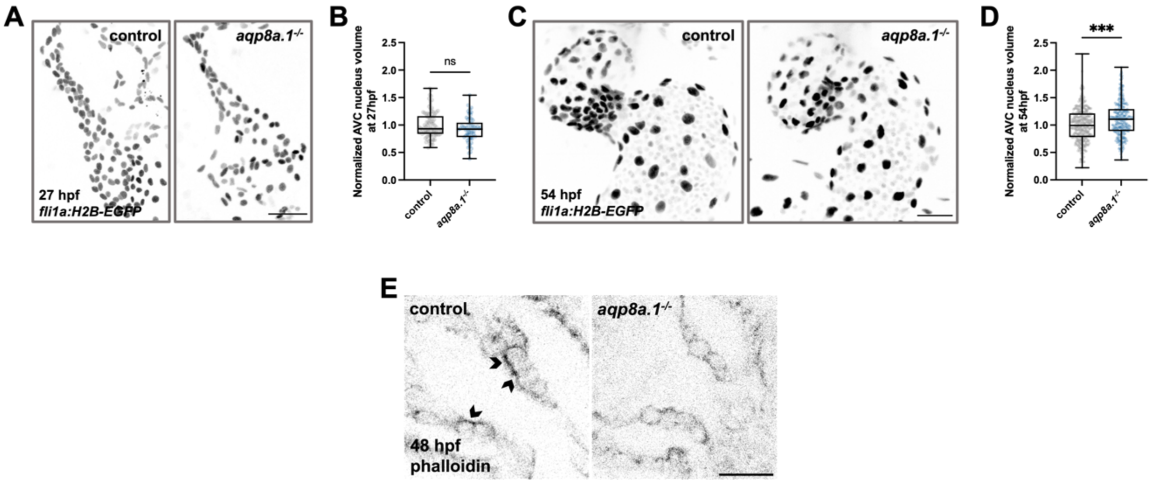

Fig. S1: Aqp8a.1 is required for the physiological reduction of AVC EdC volume. A, Representative confocal images of control and *aqp8a.1*$^{-/-}$ zebrafish hearts in *Tg(fli1a:H2B-EGFP)* background at 27 hpf. Scale bar: 40 µm. **B**, Quantification of AVC nucleus volume in control (n = 94 nuclei/14 embryos) and *aqp8a.1* single mutant (n = 59 nuclei/14 embryos) embryos in *Tg(fli1a:H2B-EGFP)* background at 27 hpf, normalized to mean control value (Mann-Whitney test; ns; two independent experiments). **C**, Representative confocal images of control and *aqp8a.1*$^{-/-}$ zebrafish hearts in *Tg(fli1a:H2B-EGFP)* background at 54 hpf. Scale bar: 40 µm. **D**, Quantification of AVC nucleus volume in control (n = 383 nuclei/24 embryos) and *aqp8a.1* single mutant (n = 277 nuclei/25 embryos) embryos in *Tg(fli1a:H2B-EGFP)* background at 54 hpf, normalized to mean control value (Mann-Whitney test; P = 0.0001; two independent experiments). **E**, Representative single z-plane immunofluorescence images of phalloidin staining in control and *aqp8a.1* mutant embryos at 48 hpf. Black arrows indicate EdCs of the AVC with enrichment in F-actin. Scale bar: 20 µm. Control in **A-D**: wild-type embryos in *Tg(fli1a:H2B-EGFP)* background; control in **E**: wild-type embryos.

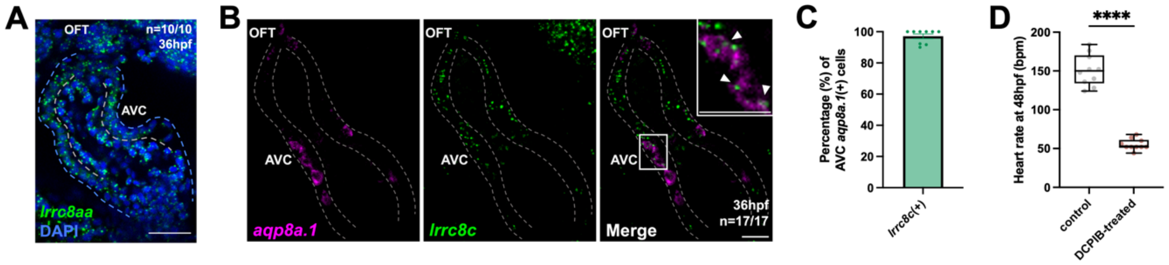

Fig. S2: *lrrc8aa* is expressed in both the endocardium and myocardium, whereas *lrrc8c* expression is primarily observed in the endocardium. **A**, Multiple z-plane projection of an RNAscope image, counterstained with DAPI, showing *lrrc8aa* mRNA distribution in the embryonic heart of wild-type zebrafish embryos (n = 10) at 36 hpf (two independent experiments). The outer blue-dotted line outlines the myocardial layer, and the inner gray-dotted line outlines the endocardial layer. Scale bar: 40 µm. **B**, Single z-plane RNAscope images showing the mRNA distribution of *aqp8a.1* and *lrrc8c* in the heart of 36 hpf wild-type embryos (n = 17) (three independent experiments). Dotted lines indicate the endocardium. Scale bars: 20 µm. **C**, Quantification of the percentage of AVC *aqp8a.1*-expressing cells [*aqp8a.1*(+)] that co-express *lrrc8c* [lrrc8c(+)] in wild-type embryos (n = 9) at 36 hpf (three independent experiments). Data are presented as mean ± SEM. **D**, Quantification of heart rate in beats per minute (bpm) in control (n = 10) and DCPIB-treated (n = 10) embryos in *Tg(kdrl:NLS-mCherry)* background at 48 hpf, based on counting the number of beats per minute at room temperature (unpaired t-test; P < 0.0001; one experiment, three independent clutches). Control in **D**: ethanol-treated embryos in *Tg(kdrl:NLS-mCherry)* background.

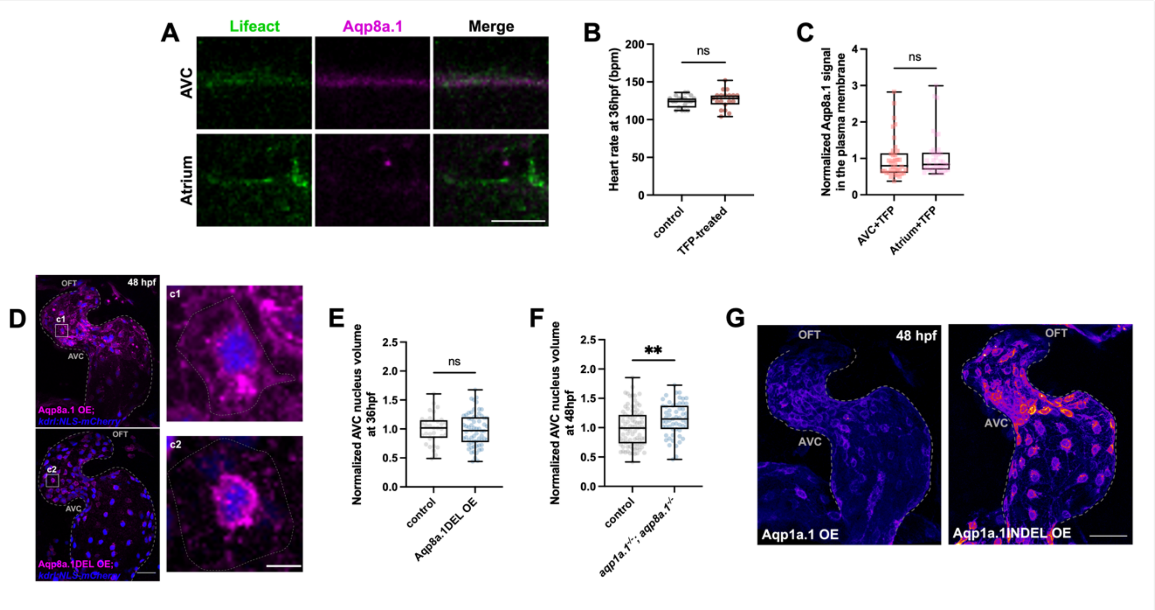

Fig. S3: The calmodulin-binding motif is a critical determinant of aquaporin plasma membrane incorporation in EdCs. **A**, Single z-plane confocal images showing plasma membrane regions, marked by Lifeact, in AVC and atrial EdCs of a 48 hpf *Tg(fli1ep:aqp8a.1-mCherry);(kdrl:Lifeact-EGFP)* heart. Scale bar: 5 µm. **B**, Heart rate in bpm in 36 hpf control (n = 19) and TFP-treated (n = 19) embryos in *Tg(kdrl:NLS-mCherry)* background, based on counting beats per minute at room temperature (unpaired t-test; ns; two independent experiments). **C**, Aqp8a.1 signal at the plasma membrane of AVC (n = 38 ROIs/6 embryos) and atrial (n = 38 ROIs/6 embryos) EdCs in 48 hpf *Tg(fli1ep:aqp8a.1-mCherry);Tg(kdrl:Lifeact-EGFP)* TFP-treated embryos, normalized to mean AVC+TFP (Mann-Whitney test; ns; one experiment, three independent clutches). **D**, Confocal images of 48 hpf Aqp8a.1 OE and Aqp8a.1DEL OE hearts. Dotted lines outline the endocardium (whole-heart images) and plasma membrane boundaries of EdCs (insets). Scale bars: 30 µm; 5 µm (inset). **E**, AVC nucleus volume in 36 hpf control (n = 45 nuclei/4 embryos) and Aqp8a.1DEL OE (n = 64 nuclei/7 embryos) embryos in *Tg(kdrl:NLS-mCherry)* background, normalized to control (unpaired t-test; ns; one experiment, three independent clutches). **F**, AVC nucleus volume in 48 hpf control (n = 91 nuclei/7 embryos) and *aqp8a.1*$^{-/-}$;*aqp1a.1*$^{-/-}$ (n = 57 nuclei/5 embryos) embryos in *Tg(fli1a:H2B-EGFP)* background, normalized to control (Mann-Whitney test; P = 0.0031; two independent experiments). **G**, Confocal images of 48 hpf Aqp1a.1 OE and Aqp1a.1INDEL OE hearts. Dotted lines outline the endocardium. Scale bar: 30 µm. Control in **B**: dH_2O-treated embryos in *Tg(kdrl:NLS-mCherry)* background; control in **E**: wild-type embryos in *Tg(kdrl:NLS-mCherry)* background; control in **F**: wild-type embryos in *Tg(fli1a:H2B-EGFP)* background. The 48 hpf wild-type dataset shown in **F** is the same in Fig. 1D. The images shown in **D** and **G** are the same in Fig. 4D and Fig. 5A, respectively.

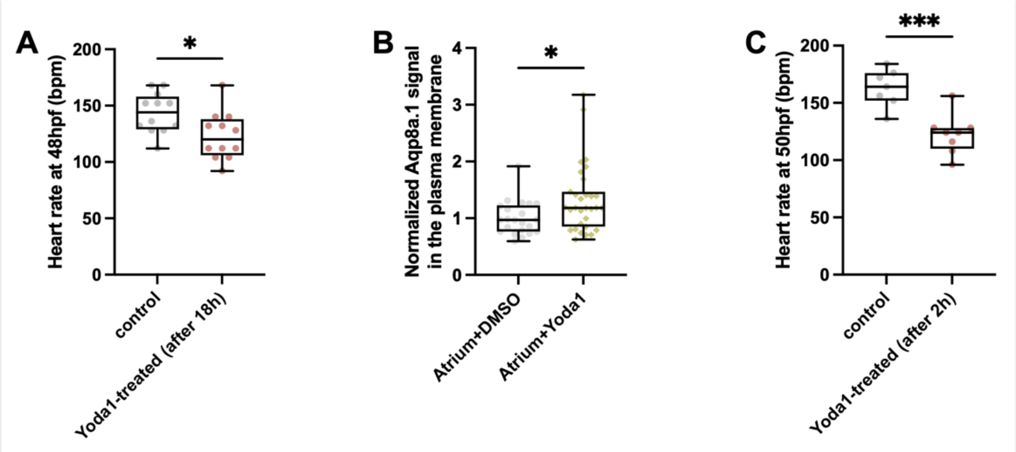

Fig. S4: Yoda1 treatment enhances Aqp8a.1 plasma membrane localization in atrial EdCs. A, Quantification of heart rate in bpm in control (n = 12 embryos) and Yoda1-treated (n = 12 embryos) embryos in *Tg(kdrl:NLS-mCherry)* background at 48 hpf, following an 18-hour incubation. Heart rate was measured by counting beats per minute at room temperature (unpaired t-test; P = 0.0178; one experiment, three independent clutches). **B**, Quantification of Aqp8a.1 signal at the plasma membrane of atrial EdCs in *Tg(fli1ep:aqp8a.1-mCherry);(kdrl:Lifeact-EGFP)* DMSO-treated (n = 22 ROIs/4 embryos) and Yoda1-treated (n = 31 ROIs/5 embryos) embryos at 48 hpf, normalized to mean Atrium+DMSO value (Mann-Whitney test; P = 0.0271; one experiment, three independent clutches). **C**, Quantification of heart rate in bpm in control (n = 7 embryos) and Yoda1-treated (n = 8 embryos) embryos in *Tg(kdrl:NLS-mCherry)* background at 50 hpf, following a 2-hour incubation. Heart rate was measured by counting beats per minute at room temperature (unpaired t-test; P = 0.0005; one experiment, two independent clutches). Control in **A**, **C**: DMSO-treated embryos in the *Tg(kdrl:NLS-mCherry)* background.

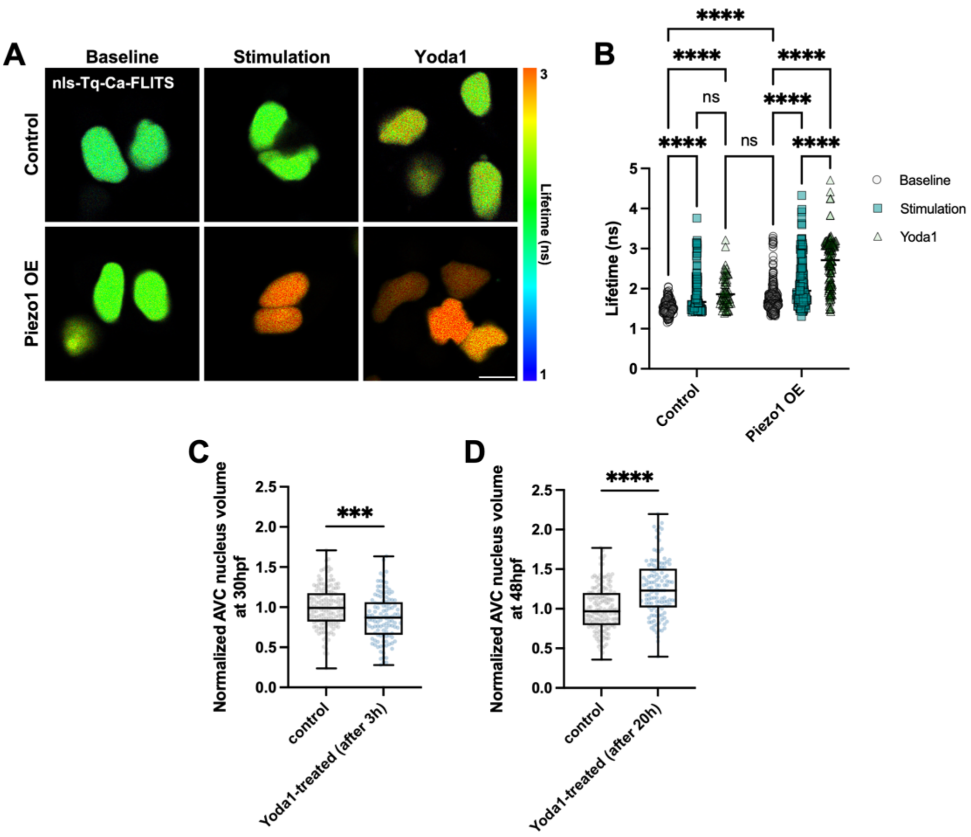

Fig. S5: Yoda1 treatment mimics the effects of Piezo1 overexpression on AVC EdC volume. A, Representative lifetime images of XLGenEPi HEK293T stable cells transiently expressing the nls-Tq-Ca-FLITS calcium biosensor, showing differences in intracellular calcium levels between control and Piezo1-overexpressing (OE) cells under baseline conditions, shear stress, and Piezo1 activation with 10 µM Yoda1. **B**, Quantification of lifetime measurements of the nls-Tq-Ca-FLITS biosensor in XLGenEPi HEK293T stable cells. Sample sizes: control baseline (n = 204 nuclei), control+shear stress (n = 102 nuclei), control+Yoda1 (n = 66 nuclei), Piezo1 OE (n = 253 nuclei), Piezo1 OE+shear stress (n = 129 nuclei), and Piezo1 OE+Yoda1 (n = 144 nuclei) (two-way ANOVA; all $P < 0.0001$; one experiment, two independent biological replicates). **C**, Quantification of AVC nucleus volume in control (n = 146 nuclei/21 embryos) and Yoda1-treated (n = 115 nuclei/18 embryos) embryos in *Tg(kdrl:NLS-mCherry)* background at 30 hpf, normalized to mean control value (unpaired t-test; $P = 0.0001$; two independent experiments). **D**, Quantification of AVC nucleus volume in control (n = 162 nuclei/12 embryos) and Yoda1-treated (n = 123 nuclei/9 embryos) embryos in *Tg(kdrl:NLS-mCherry)* background at 48 hpf, normalized to mean control value (unpaired t-test; $P < 0.0001$; one experiment, three independent clutches). Control in **A**, **B**: XLGenEPi HEK293T stable cells without doxycycline induction; control in **C**, **D**: DMSO-treated embryos in *Tg(kdrl:NLS-mCherry)* background.

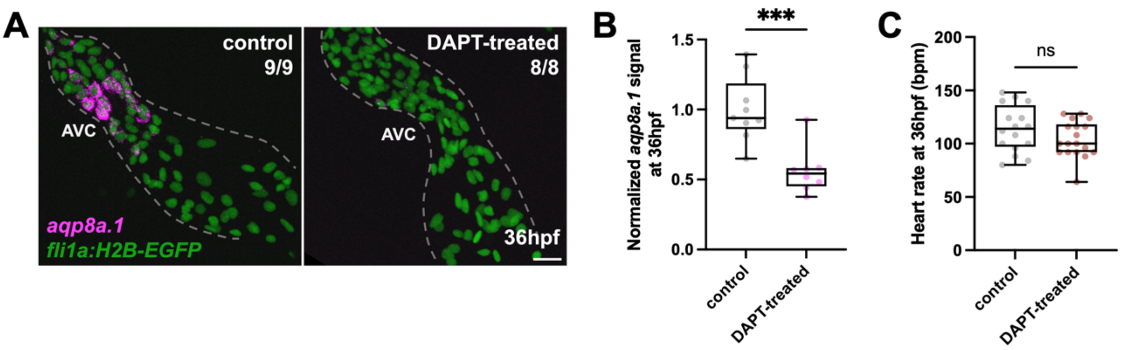

Fig. S6: Notch signaling controls the expression of *aqp8a.1*. **A**, Maximum projections of RNAscope images showing the mRNA distribution of *aqp8a.1* in the embryonic heart of control (n = 9) and DAPT-treated (n = 8) embryos in *Tg(fli1a:H2B-EGFP)* background at 36 hpf (two independent experiments). Dotted lines indicate the endocardium. Scale bar: 20 µm. **B**, Quantification of AVC RNAscope fluorescent signal based on the data shown in **A,** normalized to mean control value (unpaired t-test; P = 0.0005; two independent experiments). **C**, Quantification of heart rate in bpm in control (n = 16) and DAPT-treated (n = 18) embryos in *Tg(fli1a:H2B-EGFP)* background at 36 hpf, based on counting the number of beats per minute at room temperature (unpaired t-test; ns; two independent experiments). Control in **A**-**C**: DMSO-treated embryos in *Tg(fli1a:H2B-EGFP)* background.

Table S1: Primers for site-directed mutagenesis.

PCR mutagenesis	Forward primer (5'>3')	Reverse primer (5'>3')
aqp8a.1$^{\Delta 235}$	TCAGGAGATCCACCGGTC	TCCAACCCAATAAATCCAGTG
aqp1a.1^{Indel}	TTGGTAATGGGTGACAAGAAGGTCCGTGTTATTTTCAAAGGTGGCTCTGGAGGTGTG	TCTCACAATGCTGACAGTGACCAGAGCGCCGGTAAGAGGTCGCACACGCTCAGGGAAATC
